# FGFR2 amplification is predictive of sensitivity to regorafenib in gastric and colorectal cancers *in vitro*

**DOI:** 10.1002/1878-0261.12194

**Published:** 2018-05-29

**Authors:** Yongjun Cha, Hwang‐Phill Kim, Yoojoo Lim, Sae‐Won Han, Sang‐Hyun Song, Tae‐You Kim

**Affiliations:** ^1^ Seoul National University College of Medicine Korea; ^2^ Cancer Research Institute Seoul National University Korea; ^3^ Department of Internal Medicine Seoul National University Hospital Korea; ^4^ Department of Molecular Medicine & Biopharmaceutical Sciences Graduate School of Convergence Science and Technology Seoul National University Korea

**Keywords:** colorectal cancer, FGFR2 amplification, gastric cancer, regorafenib

## Abstract

Although regorafenib has demonstrated survival benefits in patients with metastatic colorectal and gastrointestinal stromal tumors, no proven biomarker has been identified for predicting sensitivity to regorafenib. Here, we investigated preclinical activity of regorafenib in gastric and colorectal cancer cells to identify genetic alterations associated with sensitivity to regorafenib. Mutation profiles and copy number assays of regorafenib target molecules indicated that amplification of fibroblast growth factor receptor 2 (FGFR2) was the only genetic alteration associated with *in vitro* sensitivity to regorafenib. Regorafenib effectively inhibited phosphorylation of FGFR2 and its downstream signaling molecules in a dose‐dependent manner and selectively in FGFR2‐amplified cells. Regorafenib induced G1 arrest (SNU‐16, KATO‐III) and apoptosis (NCI‐H716); however, no significant changes were seen in cell lines without FGFR2 amplification. In SNU‐16 mice xenografts, regorafenib significantly inhibited tumor growth, proliferation, and FGFR signaling compared to treatment with control vehicle. Regorafenib effectively abrogates activated FGFR2 signaling in FGFR2‐amplified gastric and colorectal cancer and, therefore, might be considered for integration into treatment in patients with FGFR2‐amplified gastric and colorectal cancers.

**Abbreviations**
FGFR2: fibroblast growth factor receptor 2
MAPK: mitogen‐activated protein kinase
MTT: 3‐(4,5‐dimethylthiazol‐2‐yl)‐2,5‐diphenyltetrazolium bromide

## 1 Introduction

Regorafenib (BAY 73‐4306) is an orally bioavailable, small‐molecule multikinase inhibitor of VEGFR1‐3, TIE2, PDGFR‐β, FGFR, KIT, RET, and BRAF (Miura *et al*., 2014; Wilhelm *et al*., 2011). As efficacy and safety were demonstrated in colorectal cancers and gastrointestinal stromal tumors (GISTs), regorafenib was recently approved for the treatment of patients with metastatic colorectal cancer and advanced GIST by the FDA in 2012 (Miura *et al*., 2014). Clinical trials assessing regorafenib in the treatment of gastric cancer, colorectal cancer, renal cell carcinoma, and hepatocellular carcinoma are underway as monotherapy as well as in combination with other chemotherapeutic agents such as 5‐fluorouracil, oxaliplatin, and irinotecan (Bruix *et al*., 2013; Eisen *et al*., 2012; Li *et al*., 2015; Pavlakis *et al*., 2016).

Although regorafenib has demonstrated survival benefits in patients with metastatic colorectal cancer and GIST and the antitumor activity of regorafenib has been demonstrated in a variety of preclinical models, the anticancer mechanism of regorafenib has remained unclear and there is no proven biomarker predicting sensitivity to regorafenib (Huynh *et al*., 2015; Strumberg *et al*., 2012). As regorafenib potentially inhibits tumor growth through anti‐angiogenesis, several angiokinases such as VEGFR1/2, PDGFR‐β, and FGFR1 are believed to be major targets of regorafenib in cancer treatment (Mross *et al*., 2012). In addition to inhibition of tumor angiogenesis, the antitumor activity of regorafenib may also involve suppression of cell proliferation and induction of apoptosis (Chen *et al*., 2014; Tai *et al*., 2014). However, it is unclear whether any of these effects is essential for the antitumor activity of regorafenib, and another potential mechanism underlying regorafenib sensitivity might further expand its indication into other types of cancers.

Therefore, we investigated the preclinical activity of regorafenib in gastric and colorectal cancer cell lines and a xenograft model to identify genetic alterations associated with sensitivity to regorafenib.

## 2 Materials and methods

### 2.1 Reagents

Regorafenib was kindly provided by Bayer Company. Stock solutions were prepared in dimethyl sulfoxide and stored at −20 °C.

### 2.2 Cell lines and cell culture

Human gastric cancer cell lines (SNU‐1, SNU‐5, SNU‐16, SNU‐216, SNU‐484, SNU‐601, SNU‐620, SNU‐638, SNU‐668, SNU‐719, AGS, MKN‐45, NCI‐N87, KATO‐III) and colorectal cell lines (SNU‐283, SNU‐1033, SNU‐C1, SNU‐C2A, SNU‐C4, SW‐403, SW‐480, HT‐29, Colo‐205, NCI‐H716) were obtained from the Korea Cell Line Bank. Cell lines were grown in RPMI‐1640 with 10% fetal bovine serum and gentamicin (10 μg·mL^−1^) at 37 °C in a 5% CO_2_‐humidified atmosphere.

### 2.3 Growth inhibition assays

The viability of cells was assessed using MTT assays (Sigma‐Aldrich, St Louis, MO, USA). A total of 3 × 10^3^ cells were seeded in 96‐well plates, incubated for 24 h, and treated for 72 h with indicated drugs at 37 °C. Following treatment, MTT solution was added to each well and incubated for 4 h at 37 °C. The medium was then removed, and dimethyl sulfoxide was added and mixed thoroughly for 30 min at room temperature. Cell viability was determined by measuring absorbance at 540 nm using a VersaMax microplate reader (Molecular Devices, Sunnyvale, CA, USA). The concentration of drug required to inhibit cell growth by 50% was determined via interpolation from dose–response curves using calcusyn software (Biosoft, Ferguson, MO, USA). Six replicate wells were utilized for each analysis, and at least three independent experiments were conducted. The data from replicate wells are presented as the mean number of the remaining cells with 95% confidence intervals.

All experiments were performed in groups of six for each regorafenib concentration and were repeated three times.

### 2.4 Mutation and copy number assays

Mutations in 12 genes encoding regorafenib target molecules (FGFR1, FGFR2, FGFR3, FGFR4, RET, KIT, VEGFR1, VEGFR2, VEGFR3, TIE2, BRAF, and PDGFR‐β) were obtained from the Cancer Cell Line Encyclopedia (CCLE; http://portals.broadinstitute.org/ccle) and the Catalog of Somatic Mutations in Cancer (COSMIC; http://cancer.sanger.ac.uk/cosmic) databases. All mutations of the genes were then reconfirmed by Sanger sequencing. Copy number alterations were analyzed by quantitative real‐time PCR. For FGFR2 gene, TaqMan copy number assays were additionally performed to calculate exact copy numbers.

### 2.5 Western blotting

Antibodies against FGFR2, phosphorylated FGFR2 (Tyr653/654), were purchased from R&D Systems (Minneapolis, MN, USA). Antibodies against phosphorylated FRS2α (Tyr436), Akt, phosphorylated Akt (Ser473), MAPK, phosphorylated MAPK (Thr202/Tyr204), P90RSK, phosphorylated P90RSK (Ser380), cyclin D, cyclin E, p27 Kip1, p21 Waf1/Cip1, cleaved caspase‐3, and PARP were purchased from Cell Signaling Technology (Beverley, MA, USA). Antibodies against actin, cyclin A, and cyclin B were purchased from Santa Cruz Biotechnology (Santa Cruz, CA, USA). Subconfluent cells (70−80%) were used for protein analyses. Cell lysates were prepared with RIPA buffer consisting of 50 mm Tris/HCl (pH 7.5), 1% (v/v) NP‐40, 0.1% (w/v) sodium deoxycholate, 150 mm NaCl, 50 mm NaF, 1 mm sodium pyrophosphate, 1 mm sodium vanadate, 1 mm nitrophenylphosphate, 1 mm benzamidine, 0.1 mm PMSF, 0.1 mm aprotinin, 0.1 mm leupeptin and 0.1 mm pepstatin A on ice for 10 min, after which the lysates were centrifuged at 14 000 × ***g*** for 20 min. Samples containing equal amounts of protein were resolved by sodium dodecyl sulfate/polyacrylamide gel electrophoresis (SDS/PAGE) followed by transfer of the proteins onto nitrocellulose membranes. The membranes were blocked for 1 h at room temperature with 5% (w/v) skim milk and incubated overnight at 4 °C with primary antibody. After probing with secondary antibody for 1 h at room temperature, detection was performed using an enhanced chemiluminescence system (Amersham Pharmacia Biotech, Piscataway, NJ, USA).

### 2.6 Cell cycle analysis

After harvesting cells, cells were washed with PBS, fixed in 70% ethanol, and stored at −20 °C until analysis. Cells were washed with PBS, digested with RNase A (50 μg·mL^−1^) for 15 min at room temperature, and then stained with propidium iodide (50 μg·mL^−1^). The cell DNA contents (10 000 cells per experiment) were analyzed using a FACS Calibur flow cytometer (Becton Dickinson Biosciences) equipped with a modfit lt program (Verity Software House, Inc., Topsham, ME, USA).

### 2.7 Small interfering RNA knockdown

Small interfering RNA against FGFR2 was purchased from Mbiotech (Seoul, Korea). Cells were transfected with small interfering RNAs (40 nm) using Lipofectamine 2000 (Invitrogen, Carlsbad, CA, USA) in accordance with the manufacturer's instructions. The sequences of the FGFR2‐specific siRNA were 5′‐CAATAGGACAGTGCTTATT‐3′ and 5′‐CTCTCTATGTCATAGTTGA‐3′, and the sequence of the control (nonspecific) siRNA was 5′‐AATTCTCCGAACGTGTCACG‐3′. After 48 h, the cells were harvested and subjected to western blot and real‐time analysis.

### 2.8 Xenograft mouse model

All animal experiments were approved by the Institute Laboratory Animal Resources Seoul National University and Use Committee. Six‐ to 8‐week‐old female BALB/c athymic (nu+/nu+) mice were purchased from Central Lab Animal Inc. (Seoul, Korea). The initial body weight of the animals at the time of arrival was between 18 and 20 g. Mice were allowed to acclimatize to local conditions for 1 week before being injected with cancer cells. Tumors were induced by injecting SNU‐16 cells (5 × 10^6^) subcutaneously into the right flank of mice. The tumors were then measured twice a week using calipers, and the tumor volume in mm^3^ was calculated according to following formula: {((width)^2^×(height))/2}. When tumor xenograft volumes reached 100–150 mm^3^, mice were randomized into three groups. Control vehicle or regorafenib at doses ranging from 15 to 30 mg·kg^−1^ in cremophor EL/95% ethanol (50 : 50) was administered orally, once daily for several weeks up to 21 days. Mice were weighed every other day starting on the date of treatment initiation and euthanized when tumors reached ~ 1.0 cm^3^ in size. Tumors were dissected and fixed in 10% formalin and embedded in paraffin. Immmunostaining against H&E and Ki‐67 was made on 10‐μm paraffin‐embedded tumor sections.

### 2.9 Statistical analysis

Tumor volumes were compared with a one‐way anova test among three groups of mice. Each regorafenib treatment group was compared with the vehicle control group for statistical significance using Dunnett's multiple comparison test.

## 3 Results

### 3.1 FGFR2 amplification is predictive of sensitivity to regorafenib in gastric and colorectal cancers

**Figure 1:**
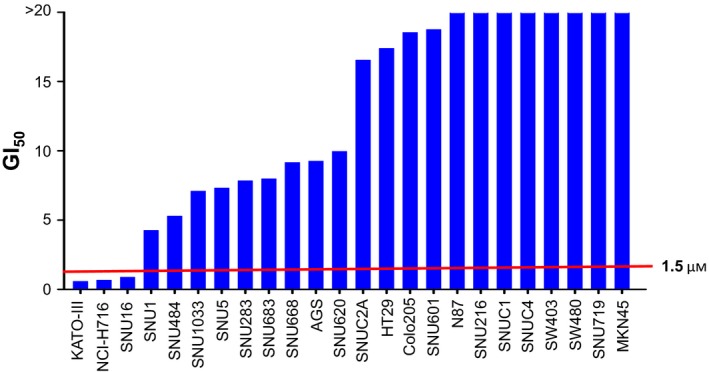
Screening of *in vitro* sensitivity to regorafenib in 14 gastric and 10 colorectal cancer cell lines. MTT cell proliferation assays were performed with increasing concentrations of regorafenib for 72 h. GI_50_ values were averaged from at least three independent experiments in hexaplicate.

We first conducted cell proliferation assays in a panel of 14 gastric and 10 colorectal cancer cell lines to identify regorafenib‐sensitive cancer cell lines of gastrointestinal tract origin (Fig. 1). In this study, regorafenib at 1.5 μm was regarded as a clinically relevant concentration for *in vitro* testing after reviewing the pharmacokinetic data of regorafenib and its metabolites (M2, M5) as well as their high protein binding (> 98%) characteristics (Rey *et al*., 2015). Cell lines with GI_50_ values less than 1.5 μm were considered sensitive while those with GI_50_ values ≥ 1.5 μm were regarded as resistant. Among the total 24 cell lines screened, two gastric (KATO‐III, SNU‐16) and one colorectal (NCI‐H716) cancer cell line showed sensitivity to regorafenib, with mean GI_50_ values ranging from 0.56 to 0.85 μm.

**Figure 2:**
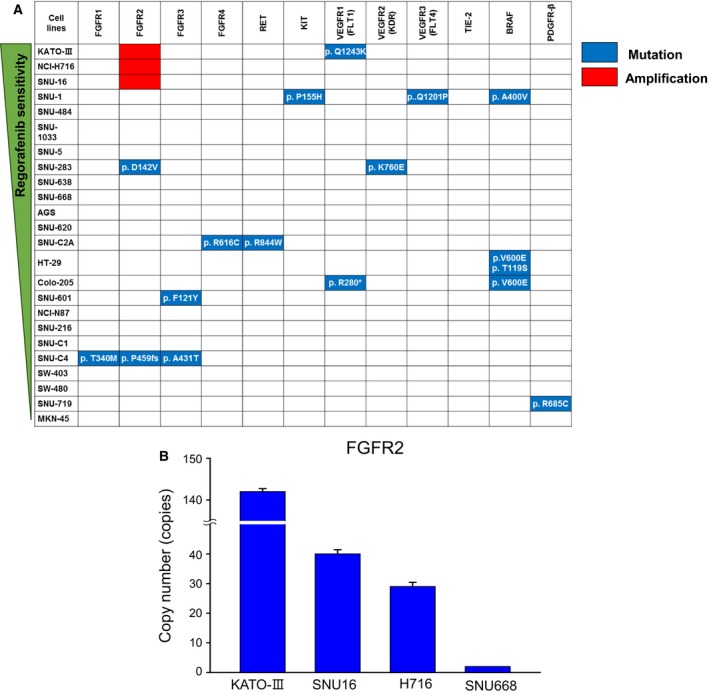
Genetic alterations of regorafenib target molecules in gastric and colorectal cancer cell lines. (A) Mutation data of 12 genes encoding regorafenib target molecules were obtained by the CCLE and COSMIC databases. (B) The TaqMan copy number assay was used to determine the copy numbers of FGFR2 in KATO‐III, SNU‐16, NCI‐H716, and SNU‐668 cells.

To identify genetic alteration(s) associated with *in vitro* sensitivity to regorafenib, we evaluated the mutation status and copy number alterations for 12 genes encoding regorafenib target molecules (FGFR1–4, RET, KIT, FLT1, KDR, FLT4, TIE‐2, BRAF, and PDGFR‐β) in all 24 cell lines (Fig. 2A). Real‐time PCR revealed amplification of FGFR2 gene in all three regorafenib‐sensitive cell lines while expression was absent in the other 21 cell lines. We did not find a relationship between amplification or deletions in the other 12 genes and regorafenib‐sensitive or regorafenib‐resistant cell lines. Likewise, presence of somatic mutations in 12 genes was not different according to regorafenib sensitivity of 24 cell lines. In addition, FBW7 mutations, which were recently suggested to be associated with resistance to regorafenib (Tong *et al*., 2017), did not show a significant relationship with differential sensitivity to the drug (Table S1). We selected one cell line (SNU‐668 cell line with KRAS Q61K mutation) among the FGFR2‐non‐amplified cell lines to be used as a control cell line in our further analyses (Fig. 2B). Copy numbers of FGFR2 gene were determined in all three regorafenib‐sensitive cell lines (KATO‐III, 143 copies; SNU‐16, 39 copies; NCI‐H716, 30 copies) and one control cell line (SNU‐668, two copies) by TaqMan copy number assays.

We additionally tested the antitumor effects of other nonselective and selective FGFR inhibitors in KATO‐III, SNU‐16, NCI‐H716, and SNU‐668 cell lines. Sorafenib, the multikinase inhibitor, and ponatinib, the pan‐FGFR inhibitor, also effectively inhibited growth of all three regorafenib‐sensitive cell lines (Table S2).

### 3.2 FGFR2‐amplified cancer cells show activated signaling through the FGFR2 pathway and are dependent on FGFR2 signaling for cellular growth

**Figure 3:**
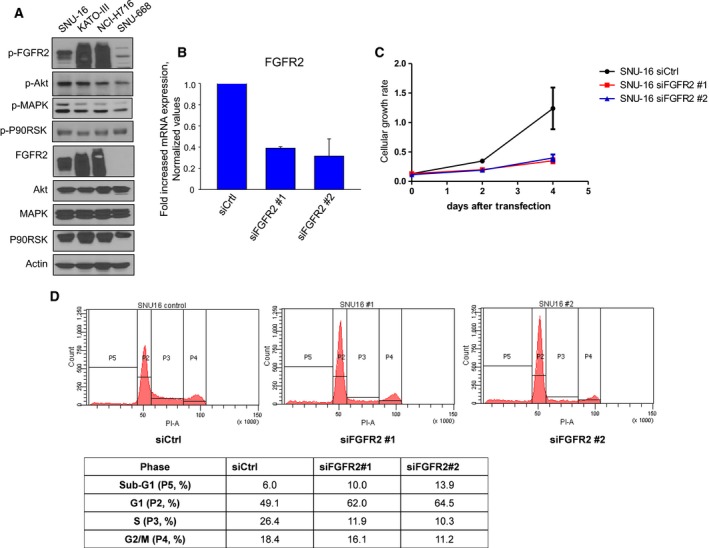
FGFR2‐amplified cancer cells show activated signaling through the FGFR2 pathway and are dependent on FGFR2 signaling for cellular growth. (A) Basal expression levels of FGFR2 signaling molecules in the indicated cell lines. (B) Knockdown of FGFR2 with siFGFR2. mRNA expression levels of FGFR2 were measured at 72 h post‐transfection in SNU‐16 cells. (C) Cell proliferation assay after knockdown of FGFR2. MTT cell proliferation assays were performed and optical density was measured daily with spectrophotometer post‐transfection in SNU‐16 cells. Values were averaged from three independent experiments in hexaplicate. (D) Cell cycle analysis after knockdown of FGFR2. FACS assays were performed at 48 h post‐transfection in SNU‐16 cells.

Western blot analysis revealed basal expression levels of FGFR2 and its phosphorylated downstream signaling molecules (p‐FGFR2, p‐Akt, p‐MAPK, and p‐P90RSK) and demonstrated that FGFR2‐amplified cell lines (SNU‐16, KATO‐III, and NCI‐H716) have activated signaling through the FGFR2 pathway compared with the non‐amplified control cell line (SNU‐668) (Fig. 3A). When FGFR2 gene was knocked down in SNU‐16 cells by small interfering RNA, cell growth rates at 48 and 96 h post‐transfection significantly decreased compared with control cells (Fig. 3B, C). Cell cycle analysis at 48 h post‐transfection demonstrated that knockdown of FGFR2 induced cell cycle arrest at G1 phase in FGFR2‐amplified cell lines (Fig. 3D).

### 3.3 Regorafenib effectively abrogates FGFR2 signaling and exhibits antitumor activity in FGFR2‐amplified cancer cells

**Figure 4:**
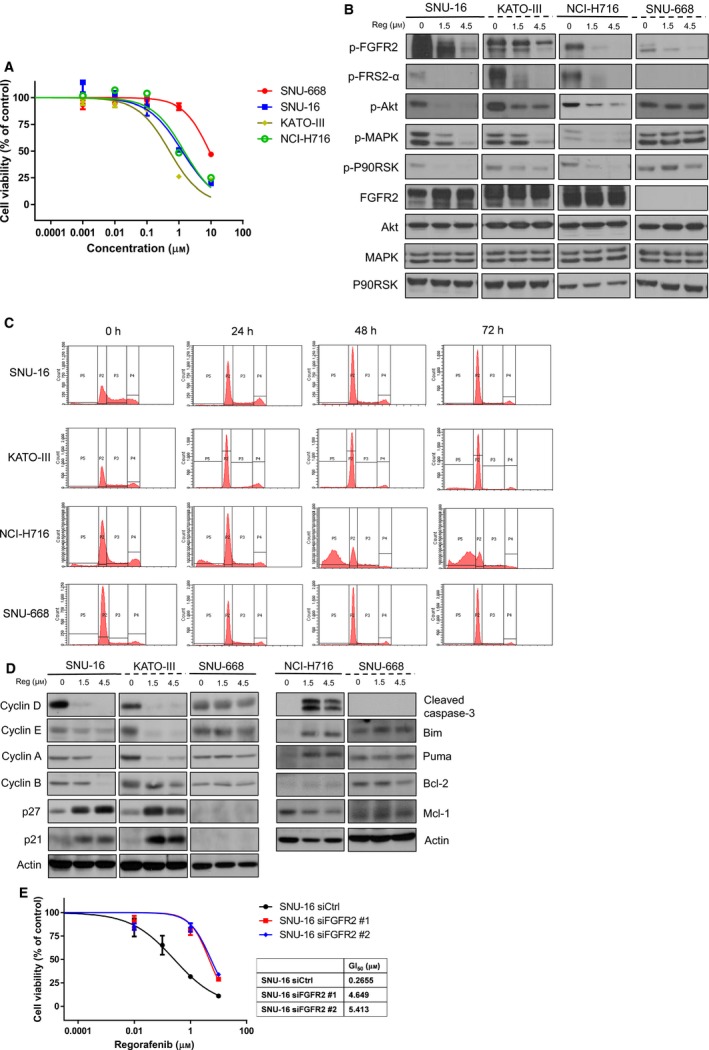
Regorafenib abrogates FGFR2 signaling and exhibits antitumor activity in FGFR2‐amplified cancer cells. (A) Cell proliferation assay of FGFR2‐amplified and FGFR2‐non‐amplified control cell lines treated with regorafenib. Cell growth curves were estimated from three independent experiments in hexaplicate. (B) Changes in FGFR2 signaling molecules after regorafenib treatment. Immunoblotting assays were performed after treatment with increasing concentrations of regorafenib for 24 h. (C) Cell cycle analysis after regorafenib. FACS assays were performed after 0–72 h after treatment with 1.5 μm regorafenib (REG). (D) Changes in cell cycle and/or apoptosis‐related molecules. (E) Cell proliferation assay of cells with knockdown of FGFR2 and regorafenib treatment. Cell growth curves and GI_50_ values were estimated from three independent experiments in hexaplicate.

Treatment with regorafenib effectively inhibited cellular growth and phosphorylation of FGFR2 and its downstream signaling molecules in a dose‐dependent manner and selectively in FGFR2‐amplified cancer cell lines (Fig. 4A, B). As GI_50_ values of regorafenib were less than 1.5 μm only for FGFR2‐amplified cells [GI_50_ in μm (mean ± SD): SNU‐16, 0.84 ± 0.77; KATO‐III, 0.56 ± 0.17; NCI‐H716, 0.83 ± 0.39; SNU‐668, 9.80 ± 2.51], regorafenib significantly inhibited cellular growth of only FGFR2‐amplified cells at its clinically relevant concentration (Fig. 4A). Immunoblotting assays also indicated that regorafenib abrogated phosphorylation of FGFR2 and downstream molecules at its clinically relevant concentration of 1.5 μm (Fig. 4B). In cell cycle analysis, regorafenib (1.5 μm) induced G1 arrest in FGFR2‐amplified gastric cell lines (SNU‐16 and KATO‐III), whereas the apoptotic cell fraction increased in NCI‐H716 colorectal cells (Fig. 4C). Changes in cell cycle and/or apoptosis‐related molecules were determined (Fig. 4D). The levels of cell proliferation‐related cyclins D, E, A, and B were all decreased after regorafenib treatment, whereas the levels of cell cycle inhibitors p21 and p27 increased after treatment in SNU‐16 and KATO‐III cells, which shows regorafenib induced G1 arrest. In addition, the expression levels of apoptosis‐regulating molecules were assessed in the NCI‐H716 cell line. The levels of the anti‐apoptotic protein MCL‐1, the pro‐apoptotic protein PUMA and the BH3‐only protein BIM were significantly altered, and caspase‐3 cleavage increased after regorafenib treatment. Notably, changes in cell cycle fractions after regorafenib treatment in SNU‐16 cells (Fig. 4C) were similar to those found after knockdown of FGFR2 gene (Fig. 3D). Cell cycle profiles did not change after regorafenib treatment in SNU‐668 control cells. We conducted cell proliferation assays with regorafenib in FGFR2‐knockdown SNU‐16 cells and found that sensitivity to regorafenib markedly decreased after knockdown of FGFR2, further supporting the idea that the antitumor activity of regorafenib is dependent on activated signaling through the FGFR2 pathway (Fig. 4E).

### 3.4 Regorafenib inhibits FGFR2 signaling and tumor growth in mice xenografts

**Figure 5:**
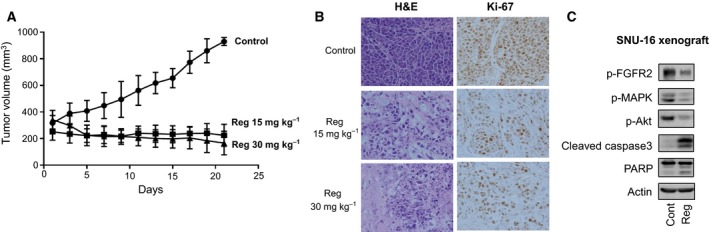
Regorafenib inhibited tumor growth of FGFR2‐amplified SNU‐16‐bearing xenografts. (A) Tumor volume changes in SNU‐16 mice xenografts after treatment with control vehicle (DMSO) or regorafenib (15 and 30 mg·kg^−1^). (B) H&E‐stained and Ki‐67‐immunostained xenograft tumor tissues harvested at day 22. (C) Changes in FGFR2 signaling and apoptosis‐related molecules after regorafenib treatment (15 mg·kg^−1^) in SNU‐16 mice xenograft.

In mice xenografts from SNU‐16 cells, oral administration of regorafenib (15 and 30 mg·kg^−1^ daily) significantly inhibited tumor growth compared to mice treated with control vehicle (Fig. 5A). Both dosage levels of regorafenib were comparable in suppressing tumor growth. Immunohistochemical staining of xenografts harvested at day 22 demonstrated that regorafenib treatment at both doses abolished tumor proliferation as determined by Ki‐67 expression level (Fig. 5B). In addition, immunoblotting assays of xenografts confirmed *in vitro* findings that regorafenib treatment significantly reduced phosphorylation of FGFR as well as its downstream molecules (Akt and MAPK), while cleavage of caspase‐3 and poly(ADP‐ribose) polymerase increased (Fig. 5C).

## 4 Discussion

In this study, we demonstrated that inhibition of FGFR signaling in FGFR2‐amplified cancers might be one of the mechanisms of antitumor activity demonstrated in preclinical and clinical evaluation of regorafenib in colorectal and gastric cancers.

FGFR signaling plays crucial roles in cancer cell proliferation, migration, angiogenesis, and survival (Dienstmann *et al*., 2014; Turner and Grose, 2010). Recent studies have uncovered increasing evidence showing that in addition to its role as an escape mechanism of anti‐VEGF therapies, deregulated FGFRs can function as driving oncogenes in certain tumor types, acting in a cell‐autonomous fashion to maintain the malignant properties of cancer cells (Knights and Cook, 2010; Korc and Friesel, 2009; Roidl *et al*., 2009). When FGFRs are mutated or amplified, aberrant activation of downstream pathways results in mitogenic, mesenchymal, and anti‐apoptotic responses in cells (Greulich and Pollock, 2011). The combination of knockdown studies and selective pharmacological inhibition in preclinical models confirms FGFRs as attractive targets for therapeutic intervention in cancer.

FGFR amplification has been reported in various cancers, including FGFR1 amplification in estrogen receptor‐positive breast cancer and squamous cell lung cancer, and FGFR2 amplification in triple negative breast cancer and gastric cancer (Wang *et al*., 2014). Recently, large‐scale genomic data including The Cancer Genome Atlas (TCGA) whole‐exome sequencing and array comprehensive genomic hybridization study identified that FGFR2 alteration is present in 9% of GC patients and that it is one of the most recurrent genetic alterations in GC (Cancer Genome Atlas Research, 2014; Deng *et al*., 2012). Notably, FGFR2 amplification mainly occurs in genomically stable cancers or cancers with chromosomal instability. The incidence of amplification is estimated to be around 5–10% among gastric cancers (Matsumoto *et al*., 2012; Su *et al*., 2014). Hence, there is significant interest in FGFR2 as a therapeutic target for FGFR2‐amplified gastric cancers, and preclinical and clinical evaluation of FGFR inhibitors are actively ongoing (Chang *et al*., 2015; Gozgit *et al*., 2012; Kim *et al*., 2014; Xie *et al*., 2013). In other types of gastrointestinal cancers including colorectal cancers, hepatocellular carcinoma, and pancreatobiliary cancers, FGFR2 amplification is not frequently demonstrated (Dieci *et al*., 2013). However, FGFR4 was recently suggested as a potential mediator of drug resistance in colorectal cancers, and signaling through FGFR might still be significant even in other types of gastrointestinal cancers (Turkington *et al*., 2014).

In our study, we demonstrated that regorafenib effectively inhibits FGFR signaling and exerts antitumor activity in FGFR2‐amplified gastric and colorectal cancer cell lines. This study has its limitation in that *in vivo* experiments were performed only for SNU‐16 cell line that successfully formed tumor in xenografts and we did not evaluate the activity of regorafenib in patients treated with the drug. However, although regorafenib is not a specific inhibitor of the FGFR2 receptor tyrosine kinase, regorafenib demonstrated antitumor activity at a clinically relevant concentration of less than 1.5 μm in this study. Currently, there is no established agent targeting FGFR2‐amplified cancers. Although preclinical data suggest antitumor activity of FGFR inhibitors in FGFR2‐amplified cancers, clinical trials evaluating efficacy and safety failed to demonstrate clinical benefits of FGFR inhibitors in FGFR2‐amplified cancers. Although regorafenib is not thought to be a more potent inhibitor of FGFR compared with specific FGFR inhibitors, regorafenib is a relatively well‐tolerated agent even in heavily treated patients (Demetri *et al*., 2013; Eisen *et al*., 2012; Grothey *et al*., 2013; Li *et al*., 2015). Regorafenib is being widely used as one of the major chemotherapeutic agents in several types of cancers. Therefore, we believe that regorafenib might be effective in patients with FGFR2‐amplified cancers and might be incorporated into treatment against this type of cancer. Clinical evaluation of regorafenib in FGFR2‐amplified colorectal and gastric cancers is warranted.

In conclusion, we demonstrated that activation of FGFR2 signaling plays a key role in colorectal and gastric cancers harboring FGFR2 amplification and regorafenib effectively blocks FGFR2 signaling and exhibits antitumor activity. Regorafenib may be a potential candidate for treatment against FGFR2‐amplified colorectal and gastric cancers.

## Conflict of interests

The authors declare that they have no conflict of interest.

## Author contributions

TYK, YC and HPK conceived the project, and TYK supervised the project. YC and HPK designed and performed *in vitro* experiments. YC and HPK analyzed the data and wrote the manuscript. YL, SWH, and SHS performed the validation. All authors reviewed the manuscript.

## Supporting information


**Table S1.** Genetic alterations of KRAS, TP53, AND FBW7 in gastric and colorectal cancer cell lines.
**Table S2.** Screening of in vitro sensitivity to sorafeniband ponatinibin KATO‐III, NCI‐H716, SNU16, and SNU668 cell lines.
