# Supplementary material for: FGFR2 amplification is predictive of sensitivity to regorafenib in gastric and colorectal cancers in vitro
Source: Mol Oncol. 2018 May 29;12(7):993–1003. doi: 10.1002/1878-0261.12194 (PMC6026866; doi:10.1002/1878-0261.12194)
Supplement: Supplementary file 1 — Table S1. Genetic alterations of KRAS, TP53, AND FBW7 in gastric and colorectal cancer cell lines. Table S2. Screening of in vitro sensitivity to sorafeniband ponatinibin KATO‐III, NCI‐H716, SNU16, and SNU668 cell lines. [file MOL2-12-993-s001.pdf]

Table S1. Genetic alterations of KRAS, TP53, AND FBW7 in gastric and colorectal cancer cell lines. Mutation data of the genes were obtained by the CCLE and COSMIC data bases

Cell lines	KRAS	TP53	FBW7
KAKO-Ⅲ			
NCI-H716		p.E224D	
SNU-16	p.R97I	p.E224D	
SNU-1	p.G12D		
SNU-484		p.G266E	
SNU-1033	p.G12D	p.R273c, p.R273H	p.G579W, p.T410T
SNU-5		p.G262*	
SNU-283			
SNU-638		p.Y282R	
SNU-668	p.Q61K	p.S215N	
AGS	p.G12D		
SNU-620		p.PSS126fs	
SNU-C2A	p.G12D	p.R273c, p.R273H, p.S185S	
HT-29		p.R273H	
Colo-205			
SNU-601	p.G12D	p.R273H	
NCI-H87			
SNU-216		p.V216M	
SNU-C1		p.S166*	
SNU-C4		p.G245S	p.R473fs
SW-403	p.G12V	p.E51*	
SW-480	p.G12V	p.R273H	
SNU-719			
MKN-45		p.R110C	

Table S2. Screening of in vitro sensitivity to sorafenib and ponatinib in KATO-Ⅲ, NCI-H716, SNU16, and SNU668 cell lines. MTT cell proliferation assays were performed with increasing concentrations of regorafenib for 72 h. GI_{50} values were averaged from at least three independent experiments in hexaplicate

Cell-lines	Sorafenib GI_{50} (μM)	Ponatinib GI_{50} (μM)
KATO-Ⅲ	0.42	0.02
NCI-H716	0.82	0.005
SNU-16	1.27	0.02
SNU-668	10.58	11.5
